# ﻿First records of two genera and thirteen species of Tabanidae (Diptera) from Honduras

**DOI:** 10.3897/zookeys.1084.77038

**Published:** 2022-01-26

**Authors:** Katerin Veroy, Jesus Orozco, Augusto L. Henriques

**Affiliations:** 1 Insect Collection, Agricultural Science and Production Department, Zamorano University (Escuela Agrícola Panamericana), Zamorano, Honduras Zamorano University Zamorano Honduras; 2 Coordenação de Pesquisas em Biodiversidade, Instituto Nacional de Pesquisas da Amazônia, Manaus, AM, Brazil Coordenação de Pesquisas em Biodiversidade, Instituto Nacional de Pesquisas da Amazônia Manaus Brazil

**Keywords:** Central America, diversity, horse flies, tabanids, taxonomy

## Abstract

This works presents information on the diversity of the Tabanidae of Honduras as a product of the examination of 386 specimens and a literature review. Thirteen species and two genera (*Bolbodimyia* and *Dasychela*) are recorded from the country for the first time. Eighty-five species distributed in 22 genera, five tribes, and three subfamilies are now known from Honduras. A key to the subfamilies, tribes, and genera of the known Honduran species is also included. All new records are mapped and illustrated to aid in the identification of the species.

## ﻿Introduction

Tabanidae is a family of Diptera that includes flies considered of medical and veterinary importance due to the blood sucking habits of the adults. Currently the group contains around 4,400 species worldwide (Pape et al. 2011). The Neotropical region has the highest diversity, with approximately 1,205 species and about 28% of the global fauna (Henriques et al. 2012), but many its areas continue to be unexplored.

The best known tabanid faunas in Central America are those of Costa Rica and Panama thanks in big part to the works of Fairchild (1961), Hogue and Fairchild (1974), Fairchild (1986), and Burger (2002). Currently, 146 species of tabanids are known from Costa Rica (Borkent et al. 2018) and 152 from Panama (Fairchild 1986). For Honduras, few works deal with the diversity of horseflies in the country, i.e., Bequaert (1925), Root (1925), and James (1950). Coscarón and Papavero (2009), in their catalog for the neotropics, listed 70 species of Tabanidae from Honduras. Henriques (2016) added two additional species, *Scionemaculipennis* (Schiner) and *Philipotabanusebrius* (Osten Sacken), for a total of 72 species.

Honduran species diversity is poorly known for many groups. Linares and Orozco (2017) estimated that at least half of the insects in the country are known unknowns, species already described that are not recorded. This poor understanding of the diversity makes conducting ecological and conservation studies very difficult in the country.

This work presents for the first time an overview of the tabanids of Honduras. By nature, this is vastly incomplete as there are many more habitats to sample and collections to revise. In comparison, Costa Rica with less than half the size of Honduras has more than twice the number of known species of tabanids. The aims of this article are: 1) to present the new findings regarding the species diversity in the country, 2) to integrate the records on the tabanid fauna of Honduras scattered in the literature, 3) to provide an updated list of the species, and 4) to create a key for the genera of tabanids known in the country.

## ﻿Methods

Material of Tabanidae deposited at the Insect Collection at Zamorano University (EAPZ) (Zamorano, Honduras) was examined. Fieldwork was done using H-traps (Egri et al. 2013), light traps, and an aerial net in several locations in Honduras. Specimens were studied under a Leica EZ4 stereo microscope using the keys provided by Bequaert (1931), Philip (1954), Fairchild and Philip (1960), Fairchild (1976), Wilkerson (1979), Fairchild (1983, 1986), Fairchild and Wilkerson (1986), Coscarón and González (1991), Burger (1996), Henriques (2006), Krolow et al. (2007), Burger (2009), Krolow and Henriques (2010), Turcatel et al. (2010), Carmo and Henriques (2019), and Turcatel (2019).

Distributional records were obtained from label data and from the literature.

A species distribution map was made for the new records using SimpleMappr (https://www.simplemappr.net/) and Microsoft Power Point v. 2112.

Photographs were taken using a Canon 100 mm lens mounted on a Canon Rebel T5i attached to a macro rail. Composite images were obtained using PICOLAY v. 2020–02–06 (http://www.picolay.de). Individual images were organized in plates in GIMP v. 2.10.24 (http://www.gimp.org).

## ﻿Results and discussion

**Figure 1.**
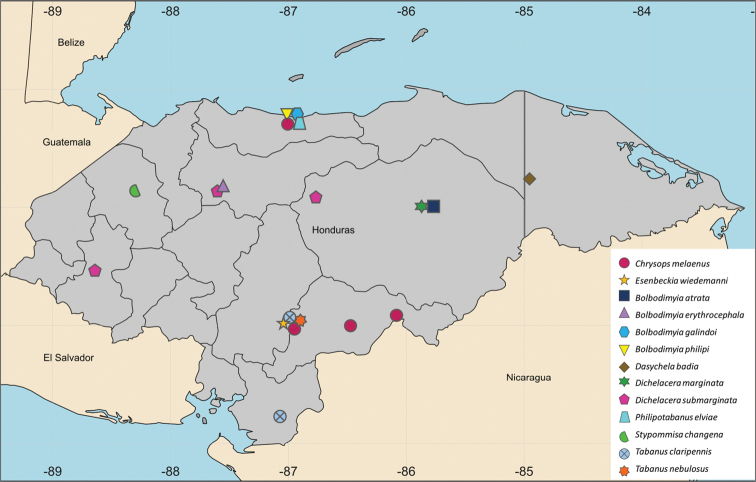
Distribution map of new records of Tabanidae from Honduras.

Eighteen genera and 47 species were found in the 386 specimens examined. Thirteen species and two genera are recorded for the first time (Fig. 1).

With these new records Honduras has now a diversity of 85 species of horseflies (Table 1). This represents an increase of 15.3% compared to the previously known taxa (72 species) but it’s still a low number, and many more species are expected to be discovered in the future. Two additional species, *Tabanusfemoralis* Kröber from Escuela Agricola Panamericana Zamorano, Francisco Morazan, and *Stypommisalerida* (Fairchild) from 15 km west of La Ceiba, Atlántida, are recorded in GBIF (https://www.gbif.org/es/occurrence/3048772282 and https://www.gbif.org/es/occurrence/3385753663). Since this material was not examined, it is not included in the list, but the records are probably valid.

**Table 1.** Species of Tabanidae from Honduras. Distributions according to Coscarón and Papavero (2009), except were indicated.

Taxon	Distribution
**CHRYSOPSINAE**	
**CHRYSOPSINI**	
*Chrysopssoror* Kröber, 1925	Guatemala, Belize, Honduras, Costa Rica, Panama, Colombia, Venezuela
*Chrysopsauroguttatus* Kröber, 1930	Mexico to Colombia
*Chrysopslatifasciatus* Bellardi, 1859	Mexico to Nicaragua
*Chrysopsmelaenus* Hine, 1925	Honduras (new record), Nicaragua, Costa Rica to Venezuela
*Chrysopsmexicanus* Kröber, 1926	Mexico to Colombia
*Chrysopspachycnemius* Hine, 1905	Mexico to Honduras
*Chrysopsscalaratus* Bellardi, 1859	Mexico to Panama
*Chrysopsvariegatus* (De Geer, 1776)	Mexico to Argentina
*Chrysopswillistoni* Hine, 1925	Mexico to Honduras
*Silviusmelanopterus* (Hine, 1905)	Mexico to Honduras
**PANGONIINAE**	
**PANGONIINI**	
*Esenbeckiaillota* (Williston, 1901)	Mexico to Honduras
*Esenbeckiamejiai* Fairchild, 1942	Guatemala to Costa Rica
*Esenbeckiaprasiniventris* (Kröber, 1929)	Guatemala to Ecuador and Trinidad, Brazil
*Esenbeckiatranslucens* (Macquart, 1846)	Mexico to Peru and Brazil
*Esenbeckiawiedemanni* (Bellardi, 1859)	Mexico, Honduras (new record)
**SCIONINI**	
*Fidenaflavipennis* Kröber, 1931	Mexico to Venezuela
*Fidenarhinophora* (Bellardi, 1859)	Mexico to Venezuela and Peru
*Scioneaurulans* (Wiedemann, 1830)	Mexico to Costa Rica
*Scionemaculipennis* (Schiner, 1868)	Honduras, Costa Rica to Venezuela, Ecuador*
**TABANINAE**	
**DIACHLORINI**	
*Bolbodimyiaatrata* (Hine, 1904)	USA, Mexico, Honduras (new record)
*Bolbodimyiaerythrocephala* (Bigot, 1892)	Honduras (new record), Costa Rica, Panama, Ecuador
*Bolbodimyiagalindoi* Fairchild, 1964	Honduras (new record), Costa Rica to Colombia
*Bolbodimyiaphilipi* Stone, 1954	Guatemala, El Salvador, Honduras (new record), Costa Rica, Panama, Colombia
*Catachloropsbaliopteru*s Gorayeb, L. Bemúdez, E.M. Bermúdez & Villalba, 1989	Mexico, Honduras, Costa Rica
*Catachloropsfulmineus* (Hine, 1920)	Honduras to Panama, Colombia, Ecuador
*Catachloropsscurrus* (Fairchild, 1958)	Mexico to Panama
*Chlorotabanusinanis* (Fabricius, 1787)	Mexico to Peru and Brazil
*Chlorotabanusmexicanus* (Linnaeus, 1758)	Mexico to Ecuador, Brazil, Trinidad
*Dasychelabadia* (Kröber, 1931)	Honduras (new record), Costa Rica, Panama
*Diachlorusferrugatus* (Fabricius, 1805)	USA to Costa Rica, Bahamas Islands
*Dichelaceracostaricana* (Fairchild, 1941)	Honduras, Costa Rica
*Dichelaceragrandis* Philip, 1943	Guatemala, Belize, Honduras
*Dichelaceramarginata* Macquart, 1847	Honduras (New record), Nicaragua to Brazil and Peru
*Dichelacerapulchroides* Fairchild & Philip, 1960	Mexico, Honduras
*Dichelaceraregina* Fairchild, 1940	Honduras to Ecuador
*Dichelacerascapularis* Macquart, 1847	Mexico to Panama
*Dichelacerasubmarginata* Lutz, 1915	Honduras (new record), Costa Rica to Venezuela, Peru, Bolivia
*Lepiselagacrassipes* (Fabricius, 1805)	Mexico to Argentina
*Leucotabanusexaestuans* (Linnaeus, 1758)	Mexico to Bolivia, Argentina, and Trinidad
*Leucotabanusnigriventris* Kröber, 1931	Mexico to Panama
*Phaeotabanuslongiappendiculatus* (Macquart, 1855)	Mexico to Panama
*Philipotabanusebrius* (Osten Sacken, 1886)	Honduras, Costa Rica, Panama*
*Philipotabanuselviae* (Fairchild, 1943)	Honduras (new record), Costa Rica, Panama
*Philipotabanuskompi* (Fairchild, 1943)	Belize, Honduras
*Philipotabanusmagnificus* (Kröber, 1934)	Honduras to Venezuela and Ecuador
*Philipotabanusnigrinubilus* (Fairchild, 1953)	Honduras, Costa Rica, Panama, Colombia, Ecuador
*Philipotabanusplenus* (Hine, 1907)	Guatemala to Colombia
*Rhabdotylusvenenatum* (Osten Sacken, 1886)	Guatemala to Ecuador
*Selasomatibiale* (Fabricius, 1805)	Mexico to Argentina
*Stenotabanusfulvistriatus* (Hine, 1912)	Mexico to Panama
*Stenotabanuslittoreus* (Hine, 1907)	Mexico to Panama
*Stenotabanusmaculifrons* (Hine, 1907)	Honduras, Costa Rica, Panama, Trinidad, Venezuela.
*Stibasomachionostigma* (Osten Sacken, 1886)	Mexico to Colombia
*Stibasomaflaviventris* (Macquart, 1848)	Mexico to Brazil
*Stibasomapanamense* Curran, 1934	Honduras to Ecuador and Venezuela
*Stypommisacaptiroptera* (Kröber, 1930)	Mexico to Guyana, Brazil, Paraguay
*Stypommisachangena* Fairchild, 1986	Honduras (new record), Costa Rica, Panama
*Stypommisau-nigrum* Philip, 1977	Mexico, Guatemala, Honduras
**TABANINI**	
*Poeciloderasquadripunctatus* (Fabricius, 1805)	Mexico to Argentina
*Tabanusabattenuis* Philip, 1969	Mexico, Guatemala, El Salvador, Honduras, Nicaragua
*Tabanusbigoti* Bellardi, 1859	Mexico to Colombia and Venezuela
*Tabanusclaripennis* (Bigot, 1892)	Honduras (new record), West Indies, Costa Rica to Paraguay, Brazil, Argentina, and Chile
*Tabanuscolombensis* Macquart, 1846	USA to Trinidad, Venezuela, Ecuador, Brazil
*Tabanuscommixtus* Walker, 1860	Mexico to Venezuela, Hispaniola, Trinidad, Martinique
*Tabanusdefilippii* Bellardi, 1859	Mexico to Panama
*Tabanusdorsifer* Walker, 1860	USA, Mexico, Honduras
*Tabanuserebus* Osten Sacken, 1886	Honduras, Nicaragua, Costa Rica, Panama
*Tabanusjilamensis* Hine, 1925	Honduras
*Tabanusmorbosus* Stone, 1938	USA, Mexico to Panama
*Tabanusnebulosus* De Geer, 1776	Belize, Honduras (New record), Costa Rica, Trinidad, Barbados to Brazil and Argentina
*Tabanusoccidentalis* Linnaeus, 1758	Mexico to Argentina, Trinidad
*Tabanusoculus* Walker, 1848	Mexico to Panama
*Tabanuspicturatus* Kröber, 1931	Mexico, Belize, Honduras
*Tabanuspolyphemus* Fairchild, 1958	Mexico to Colombia
*Tabanuspruinosus* Bigot, 1892	USA to Panama
*Tabanuspseudoculus* Fairchild, 1942	Guatemala to Colombia, Venezuela, Ecuador, and Trinidad
*Tabanuspungens* Wiedemann, 1828	USA, Neotropics (except West Indies and Chile), Trinidad
*Tabanusquinquepunctatus* Hine, 1925	Guatemala, Belize, Honduras, Costa Rica, Panama
*Tabanussecundus* Walker, 1848	Guatemala to Peru, Surinam, and Paraguay
*Tabanussubruber* Bellardi, 1859	Mexico, Guatemala, Honduras
*Tabanusunipunctatus* (Bigot, 1892)	Mexico to Colombia
*Tabanusunistriatus* Hine, 1906	Guatemala to Ecuador
Tabanusvittigerssp.guatemalanus Hine, 1906	USA, Bahamas, West Indies, Mexico to Surinam, French Guiana, and Brazil
*Tabanusxenorhynchus* Fairchild, 1947	Guatemala to Panama
*Tabanusyucatanus* Townsend, 1897	Mexico, Guatemala, El Salvador, Honduras, Nicaragua

* Distribution according to Henriques (2016).

### ﻿New Tabanidae from Honduras

**CHRYSOPSINAE**

#### CHRYSOPSINI

##### ﻿ Chrysops melaenus

Taxon classificationAnimaliaDipteraTabanidae

Hine, 1925

Figure 2A

###### Distribution.

Previously known from Nicaragua to Venezuela (Coscarón and Papavero 2009).

**Figure 2.**
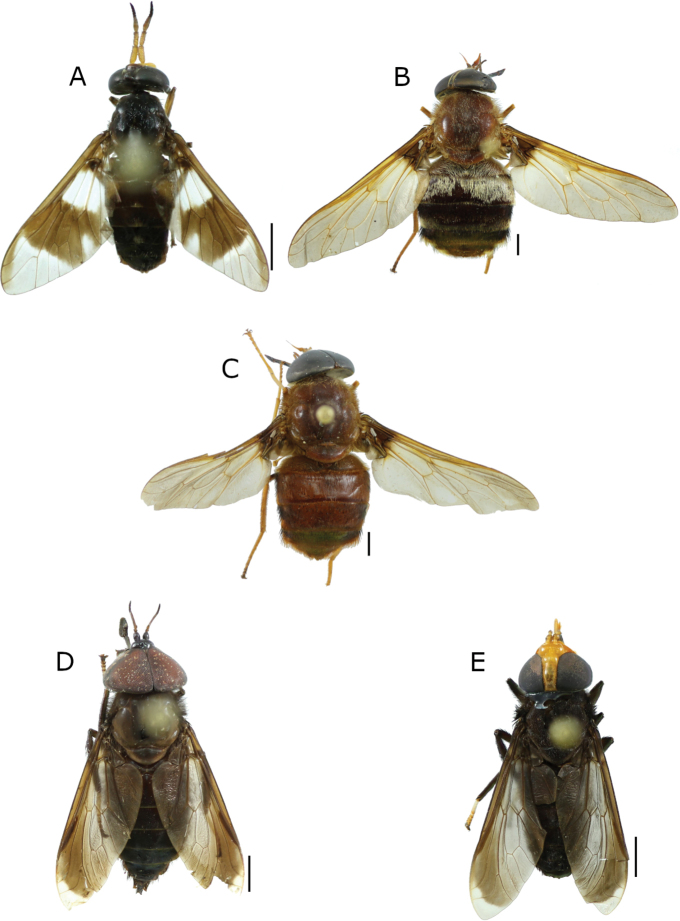
New records of Tabanidae from Honduras **A***Chrysopsmelaenus* Hine (♀) **B, C***Esenbeckiawiedemanni* (Bellardi) (♀, ♂) **D***Bolbodimyiaatrata* (Hine) (♂) **E***B.erythrocephala* (Bigot) (♀). Scale bars: 2 mm.

###### Material examined.

Honduras: 1♂, Atlántida, RVS Cuero y Salado, Salado Barra, 15°46'02"N, 86°59'51"W, 2 m, 25.i.2000, R. Cave, R. Cordero and J. Torres leg.; EAPZ22.445. 1♂, El Paraíso, 5.3 km N Cifuentes, 14°05'48"N, 86°06'57"W, 13.vi.1999, R. Cave and J. Torres leg.; EAPZ69.749. 1♀, El Paraíso, Danlí, Cerro Apaguiz 14°00'27"N, 86°32'26"W, 20.ii.1988, R. Cordero leg.; EAPZ42.723. 1♀, Francisco Morazán, 32 km Tegucigalpa, El Zamorano, 14°01'N, 87°00'W , J. Cabezas leg.; EAPZ42.698.

#### ﻿PANGONIINAE

**PANGONIINI**

##### ﻿ Esenbeckia wiedemanni

Taxon classificationAnimaliaDipteraStratiomyidae

(Bellardi, 1859)

Figure 2B, C

###### Distribution.

Previously known exclusively from Mexico (Coscarón and Papavero 2009).

###### Material examined.

Honduras: 1♂, 1♀, Francisco Morazán, Masicarán, Uyúca, 14°01'00"N, 87°05'00"W, 10–15.xi.2016, E. van den Berghe leg.; EAPZ42.764.

#### ﻿TABANINAE

**DIACHLORINI**

##### ﻿ Bolbodimyia atrata

Taxon classificationAnimaliaDipteraTabanidae

(Hine, 1904)

Figure 2D

###### Distribution.

Previously known from U.S.A. and Mexico (Coscarón and Papavero 2009).

###### Material examined.

Honduras: 2♂♂, Olancho, El Murmullo, Sierra de Agalta, 15°01'00"N, 85°47'00"W, 28.vi.1997, R. Cave leg.; EAPZ69.815.

##### ﻿ Bolbodimyia erythrocephala

Taxon classificationAnimaliaDipteraTabanidae

(Bigot, 1892)

Figure 2E

###### Distribution.

Previously known from Costa Rica, Panama, Ecuador (Coscarón and Papavero 2009), and Colombia (Wolff and Miranda-Esquivel 2016).

###### Material examined.

Honduras: 1♀, Yoro, Par. Nac. Pico Pijol, 15°13'00"N, 87°33'00"W, 22–23.vi.1998, R. Cave leg.; EAPZ42.652.

##### ﻿ Bolbodimyia galindoi

Taxon classificationAnimaliaDipteraTabanidae

Fairchild, 1964

Figure 3A, B

###### Distribution.

Previously known from Costa Rica to Colombia (Coscarón and Papavero 2009).

**Figure 3.**
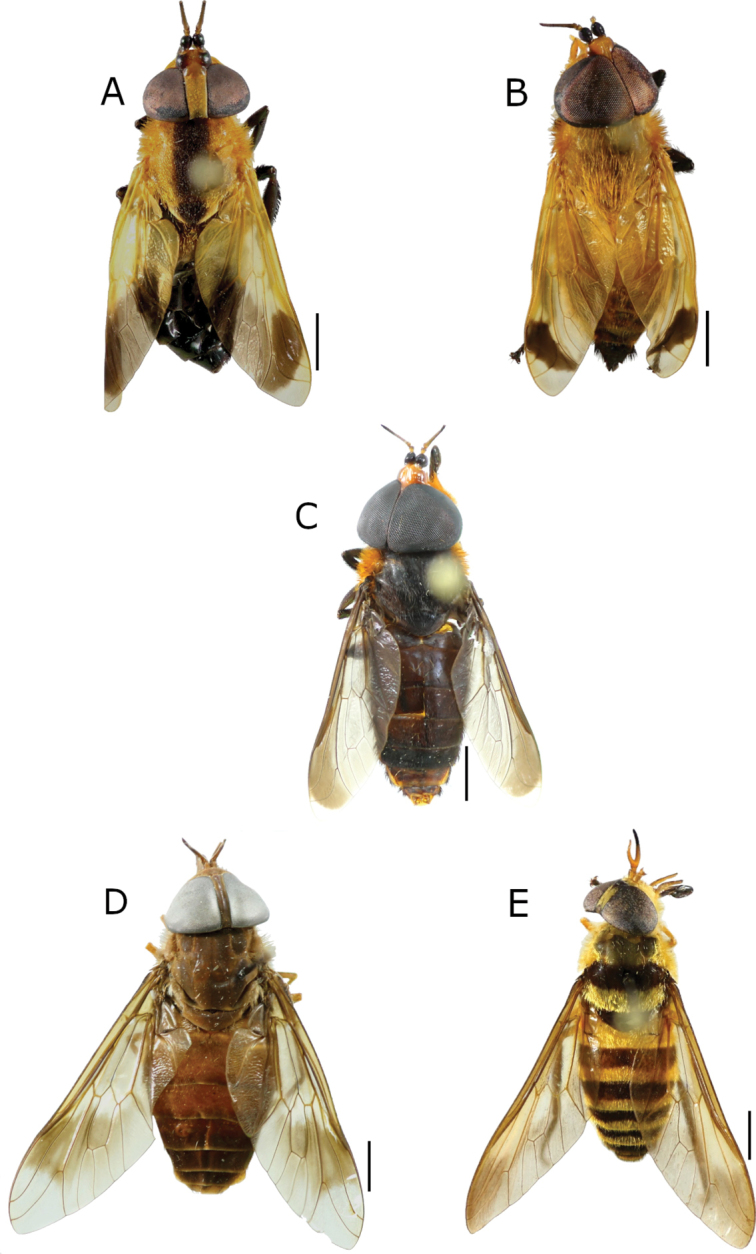
New records of Tabanidae from Honduras. **A, B***Bolbodimyiagalindoi* Fairchild (♀, ♂) **C***B.philipi* Stone (♂) **D***Dasychelabadia* (Kröber) (♀) **E***Dichelaceramarginata* Macquart (♀). Scale bars: 2 mm.

###### Material examined.

Honduras: 1♂, 1♀, Atlántida, Par. Nac. Pico Bonito, Rio Zacate, 15°41'35"N, 86°55'58"W, 35 m, 5.iii.2000, R. Cave, R. Cordero and J. Torres leg.; EAPZ27.180.

##### ﻿ Bolbodimyia philipi

Taxon classificationAnimaliaDipteraTabanidae

Stone, 1954

Figure 3C

###### Distribution.

Previously known from Guatemala, El Salvador, Costa Rica, Panama, and Colombia (Coscarón and Papavero 2009).

###### Material examined.

Honduras: 1♂, Atlántida, Cuero y Salado, Salado Barra, 15°46'02"N, 86°59'51"W, 2 m, 25.i.2000, R. Cave, R. Cordero and J. Torres leg.; EAPZ22.452.

##### ﻿ Dasychela badia

Taxon classificationAnimaliaDipteraTabanidae

(Kröber, 1931)

Figure 3D

###### Distribution.

Previously known from Costa Rica and Panama (Coscarón and Papavero 2009).

###### Material examined.

Honduras: 23♀♀, Gracias a Dios, Ciudad Blanca, 15°14'47"N, 84°58'2"W, 250 m, 15–26.ii.2017, E. van den Berghe leg., light trap; EAPZ43.577.

##### ﻿ Dichelacera marginata

Taxon classificationAnimaliaDipteraTabanidae

Macquart, 1847

Figure 3E

###### Distribution.

Previously known from Nicaragua to Brazil and Peru (Coscarón and Papavero 2009).

###### Material examined.

Honduras: 1♀, Olancho, El Murmullo, Sierra de Agalta, 15°01'00"N, 85°47'00"W, 28.vi.1997, R. Cave leg.; EAPZ44.214.

##### ﻿ Dichelacera submarginata

Taxon classificationAnimaliaDipteraTabanidae

Lutz, 1915

Figure 4A, B

###### Distribution.

Previously known from Costa Rica to Venezuela, Peru, and Bolivia (Coscarón and Papavero 2009).

**Figure 4.**
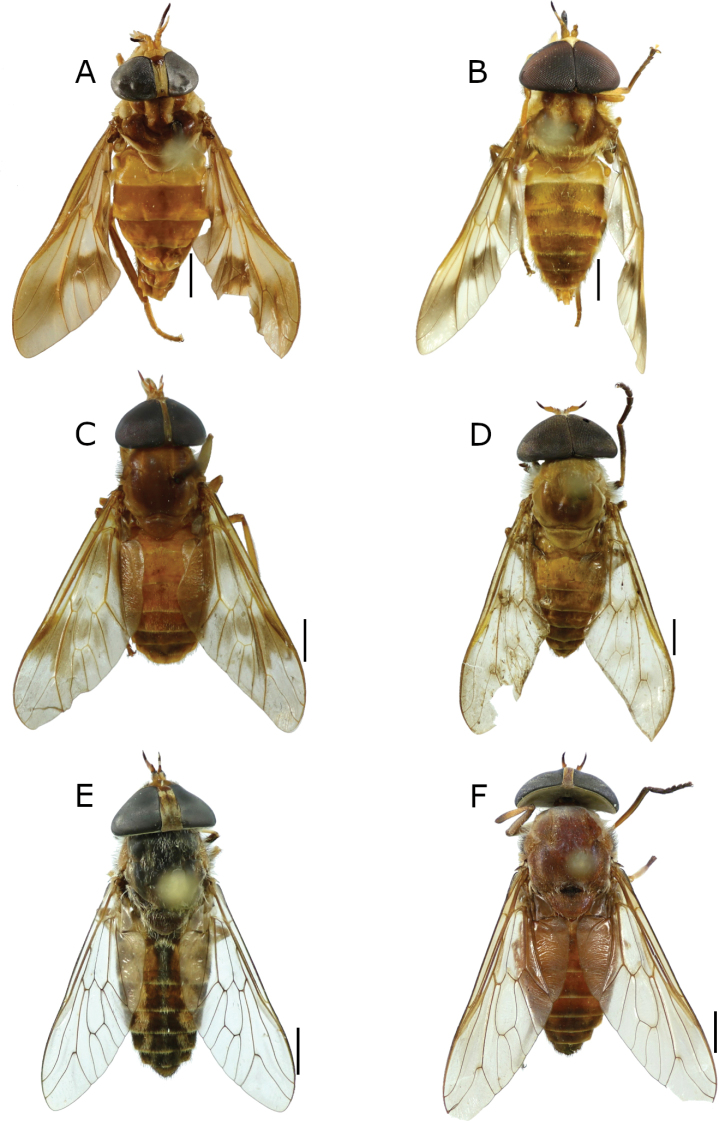
New records of Tabanidae from Honduras **A, B***Dichelacerasubmarginata* Lutz (♀, ♂) **C***Philipotabanuselviae* (Fairchild) (♀) **D***Stypommisachangena* Fairchild (♂) **E***Tabanusclaripennis* (Bigot) (♀) **F***T.nebulosus* De Geer (♀). Scale bars: 2 mm.

###### Material examined.

Honduras: 1♀, Olancho, La Muralla, 15°04'56"N, 86°45'24"W, 26–30.iii.2013, O. Schlein leg.; EAPZ42.549. 1♂, Lempira, Par. Nac. Celaque, 14°28'46"N, 88°38'35"W, 1400 m, 27.iv.2018, E. van den Berghe leg.; EAPZ69.831. 1♂, Yoro, Par. Nac. Pico Pijol, Linda Vista, 15°10'35"N, 87°35'10"W, 1450 m, 21.iv.1999, R. Cave and J. Torres leg.; EAPZ42.829.

##### ﻿ Philipotabanus elviae

Taxon classificationAnimaliaDipteraTabanidae

(Fairchild, 1943)

Figure 4C

###### Distribution.

Previously known from Costa Rica and Panama (Coscarón and Papavero 2009).

###### Material examined.

Honduras: 12 ♀♀, Atlántida, Par. Nac. Pico Bonito, Rio Zacate, 15°41'35"N, 86°55'58"W, 35 m, 5.v.2000, R. Cave leg.; EAPZ29.665.

##### ﻿ Stypommisa changena

Taxon classificationAnimaliaDipteraTabanidae

Fairchild, 1986

Figure 4D

###### Distribution.

Previously known from Costa Rica and Panama (Coscarón and Papavero 2009).

###### Material examined.

Honduras: 1 ♂, Santa Bárbara, El Volcán, Trinidad, 15°08'02"N, 88°18'01"W, 1320 m, 26.vi.2000. R. Cordero and J. Torres leg.; EAPZ35.149.

#### TABANINI

##### ﻿ Tabanus claripennis

Taxon classificationAnimaliaDipteraTabaninae

(Bigot, 1892)

Figure 4E

###### Distribution.

Previously known from the West Indies, Costa Rica to Paraguay, Brazil, Argentina, and Chile (Coscarón and Papavero 2009).

###### Material examined.

Honduras: 7 ♀♀, Francisco Morazán, El Zamorano, EAP, 14°01'N, 87°00'W, 5–29.vii.2020, H-trap, R. Argueta leg.; EAPZ43.572. 1♂, Choluteca, 6.7 km SE Santa Ana de Yusguare, 13°15'37"N, 87°04'40"W, 8.ix.1999, R. Cave and J. Torres leg.; EAPZ43.570.

##### ﻿ Tabanus nebulosus

Taxon classificationAnimaliaDipteraTabaninae

De Geer, 1776

Figure 4F

###### Distribution.

Previously known from Belize (Coscarón and Papavero 2009), Costa Rica (Fairchild 1961), Colombia, Venezuela, Trinidad, Surinam, Brazil, Bolivia, Paraguay, Barbados, and Argentina (Coscarón and Papavero 2009; Henriques 2016).

###### Material examined.

Honduras: 2 ♀♀, Francisco Morazán, El Zamorano EAP, 14°01'N, 87°00'W, 850 m, v–vii, Estudiante EAPZ leg.; EAPZ75.022. 1 ♀, Francisco Morazán, El Zamorano EAP, 14°01'N, 87°00'W, 850 m, 31.v.2019, L. Moreno leg.; EAPZ75.023.

### ﻿Key to the subfamilies, tribes, and genera of Tabanidae from Honduras

Modified from Fairchild (1969) and Burger (2009).

**Table:** 

1	Hind tibiae without paired terminal spurs or spines; TABANINAE	**6**
–	Hind tibiae with paired terminal spurs or spines, spines rarely absent or difficult to see	**2**
2	Third antennal segment with 7 or 8 distinct flagellomeres; tergite 9 undivided; PANGONIINAE	**3**
–	Third antennal segment with no more than 5 distinct flagellomeres; tergite 9 divided; CHRYSOPSINAE	**5**
3	Eyes bare; frons with ridge-like callus, which may be bare or tomentose; PANGONIINI	***Esenbeckia* Rondani**
–	Eyes pilose; frons flat, without any sort of callus; SCIONINI	**4**
4	Cell m_3_ closed at wing margin	***Scione* Walker**
–	Cell m_3_ open at wing margin	***Fidena* Walker**
5	Wings with dark crossband (Fig. 2A), crossband absent at times; eyes in life with pattern of dots and bars	***Chrysops* Meigen**
–	Wings hyaline or cloudy on cross veins or elsewhere, without distinct crossband; eye pattern in life irregularly speckled	***Silvius* Meigen**
6	Basicosta without strong setae, if setae present usually less dense than those on adjoining costa; if setae on basicosta as dense as on costa, then vestiges of ocelli present; DIACHLORINI	**7**
–	Basicosta with numerous strong setae, setae equal in size and density to those on adjoining costa, if setae sparse, then without vestiges of ocelli; TABANINI	**22**
7	Third antennal segment with strong dorso-basal tooth or forward-pointing spine that often reaches to or beyond end of first flagellomere	**8**
–	Third antennal segment usually at most with acute dorso-basal angle	**12**
8	Eyes densely pilose; antennal tooth reaching beyond apex of first flagellomere; proboscis longer than maxillary palpi; maxillary palpi slender, generally exceeding antennae; labella short, membranous; callus club shaped, much narrower than frons; wings with diffuse dark discal marking	***Dasychela* Enderlein**
–	Eyes bare; other characters variable	**9**
9	Stout species; body sometimes hairy and beelike; foretibiae usually inflated; long hair fringes on at least hind tibiae; maxillary palpi inflated; antennae short, stout, with dorsal tooth extending beyond apex of first flagellomere; labella shiny and sclerotized	**10**
–	Slender species; all tibiae slender; rest of characters not as above	**11**
10	Abdomen green or greenish, sparsely covered with hairs; hind tibial fringe moderate in length; all tibiae slender; wings hyaline, sometimes yellowish; not resembling bees	***Rhabdotylus* Lutz**
–	Abdomen not greenish, densely hirsute; hind tibial fringe long; at least foretibia inflated; wings variable, never entirely hyaline or uniformly tinted, generally with black or contrasting pattern; body often resembling bees (see Turcatel et al. 2010)	***Stibasoma* Schiner**
11	Basal callus thin, ridge-like, narrower than frons; eyes unicolored, bright green in life, rarely bicolored or with faint median line; mesoscutum unicolored or weakly striped, not transversely banded	***Catachlorops* Lutz**
–	Basal callus as wide as frons; eyes banded or unicolorous blackish in life; mesoscutum often transversely banded	***Dichelacera* Macquart**
12	Subcallus, and usually first antennal segment, greatly inflated and shiny; third antennal segment long and slender, with obtuse dorso-basal angle; tibiae slender or slightly incrassate; wings black or partly so, with apex sharply hyaline, apical half of vein R_4_ bent sharply forward; maxillary palpi moderately slender, tomentose; clypeus tomentose	***Bolbodimyia* Bigot**
–	Without above combination of characters	**13**
13	Tibiae, especially first two pairs, greatly inflated; subcallus, clypeus, and gena bare; maxillary palpi shiny and flattened; wings black at base, at least to ends of cells br and bm; labella membranous	**14**
–	Tibiae not or but slightly inflated; without above combination of characters	**15**
14	Large, shiny bluish-black species; wings black from base to middle of cell d	***Selasoma* Macquart**
–	Small species, mesoscutum, and often abdomen, with metallic brassy or greenish scale-like hairs; wings black from base to beyond end of cell d, with hyaline triangle in cells m_3_ and cua_1_	***Lepiselaga* Macquart**
15	Mesopleura shiny or pearly tomentose in contrast to rest of pleura; wings usually with dark subapical marking	***Diachlorus* Osten Sacken**
–	Mesopleura not shiny or pearly tomentose, not contrasting with other pleural sclerites; wings without dark subapical marking	**16**
16	Basal callus absent	***Chlorotabanus* Lutz**
–	Basal callus present, reduced at times	**17**
17	Labella sclerotized; frons narrow, generally over 5 times as long as its basal width; eyes in life unicolored, unbanded; dorsal angle on third antennal segment strong	***Phaeotabanus* Lutz**
–	Labella membranous; frons generally less than 4 times as long as its basal width; eyes in life usually banded; dorsal angle of third antennal segment variable	**18**
18	Eyes bare, with at least 2 transverse bands in life; mostly small species with moderately broad frons often with median dark-haired patch; callus rounded or square, generally as wide as frons	***Stenotabanus* Lutz**
–	Eyes pilose or bare, with at most 1 dark median, generally unicolored, rarely bicolored; rest of characters not as above	**19**
19	Vertex with well-marked tubercle and/or with clear vestiges of ocelli; eyes bare; frons narrow; basal callus club-shaped or ridge-like	**20**
–	Vertex without tubercle or clear vestiges of ocelli, slightly raised shiny or discolored tubercle rarely present; if tubercle present, then eyes pilose, or frons broad, or basal callus rounded	**22**
20	Wings with extensive dark pattern not consisting of spots on cross veins; if wings apparently unmarked, then thorax prominently striped, or frons exceedingly narrow and callus thread-like	***Philipotabanus* Fairchild**
–	Wings hyaline, tinted, or with dark pattern consisting primarily of dark spots around cross veins	**21**
21	Wings hyaline or evenly tinted, with costal cell often darker, but never with apical clouds or spots on cross veins; frontal callus clavate or ridge-like; abdomen black or brown, nearly always with transverse bands at least on fourth segment, rarely otherwise; appendix on fork of vein R_4_ absent	***Leucotabanus* Lutz**
–	Wing with clouds on at least discal cross veins, often with apical infuscation, if entirely hyaline or tinted, then abdomen and thorax not as above; frontal callus variable; wings often with appendix on fork of vein R_4_	***Stypommisa* Enderlein**
22	Vertex with small, rounded, sometimes indistinct, tubercle; eyes of female usually pilose, densely so on males; wings with all cross veins prominently spotted	***Poeciloderas* Lutz**
–	Vertex rarely with tubercle; without above combination of characters	***Tabanus* Lutz**

## Supplementary Material

XML Treatment for Chrysops melaenus

XML Treatment for Esenbeckia wiedemanni

XML Treatment for Bolbodimyia atrata

XML Treatment for Bolbodimyia erythrocephala

XML Treatment for Bolbodimyia galindoi

XML Treatment for Bolbodimyia philipi

XML Treatment for Dasychela badia

XML Treatment for Dichelacera marginata

XML Treatment for Dichelacera submarginata

XML Treatment for Philipotabanus elviae

XML Treatment for Stypommisa changena

XML Treatment for Tabanus claripennis

XML Treatment for Tabanus nebulosus
